# Primary myoepithelial carcinoma of the gallbladder: a case report and literature review

**DOI:** 10.3389/fonc.2026.1782500

**Published:** 2026-08-07

**Authors:** Chen Xu, Quanyong Wang, Fene Hao, Lin Shi, Lu Zhu

**Affiliations:** 1 Department of Radiology, Affiliated Hospital of Inner Mongolia Medical University, Huhhot, Inner Mongolia, China; 2 Department of Radiology, Inner Mongolia Autonomous Region Hospital of Traditional Chinese Medicine, Huhhot, Inner Mongolia, China; 3 Department of Pathology, Inner Mongolia Medical University, Huhhot, Inner Mongolia, China

**Keywords:** case report, differential diagnosis, gallbladder, image characteristic, myoepithelial carcinoma of soft tissue

## Abstract

Myoepithelial carcinoma (MC) is an exceptionally rare malignant neoplasm that predominantly originates in the salivary glands, with occurrence in visceral soft tissues being exceedingly sparse. Herein, we report the first documented case of primary MC of the gallbladder in a 57-year-old male. Although the patient exhibited non-specific clinical symptoms, radiological evaluations revealed distinctive features, including irregular wall thickening and progressive, non-uniform intraluminal enhancement. Histopathological and immunohistochemical analyses confirmed the diagnosis. Furthermore, we conducted a comprehensive review of the relevant literature, with a specific focus on the imaging features of MC. By consolidating these findings, this study aims to enhance clinical recognition, refine diagnostic accuracy, and provide a valuable reference for the management of this rare malignancy.

## Introduction

Myoepithelial carcinoma (MC), alternatively termed malignant myoepithelioma, is a rare malignancy that predominantly originates in the salivary glands, particularly the parotid gland. Its incidence remains extremely low, representing approximately 0.2% to 0.6% of all salivary gland tumors (1). Primary MC originating in visceral soft tissues is exceptionally rare. This tumor can occur at any age but is most commonly observed in young and middle-aged adults, with a median age of 40 years (2, 3).

Herein, we present the first documented case of primary MC of the gallbladder and summarize its imaging features. This report aims to delineate its distinctive radiological hallmarks and conduct a comprehensive review of the relevant literature to enhance clinical recognition of this rare entity. We present this case in accordance with the CARE reporting checklist.

## Case report

A 57-year-old male was incidentally diagnosed with cholelithiasis during a routine physical examination in February 2023. The patient reported intermittent upper abdominal pain that was resolved spontaneously. His medical history was notable for hypertension, which was well-controlled through consistent antihypertensive therapy. He was admitted to the Affiliated Hospital of Inner Mongolia Medical University for definitive surgical management in October 2023. Laboratory tests revealed an elevated carbohydrate antigen 19-9 (CA19-9) level of 800.76 U/mL, a ferritin level of 1113.98 ng/mL, and an increased urinary amylase level of 180 IU/mL.

**Figure 1:**
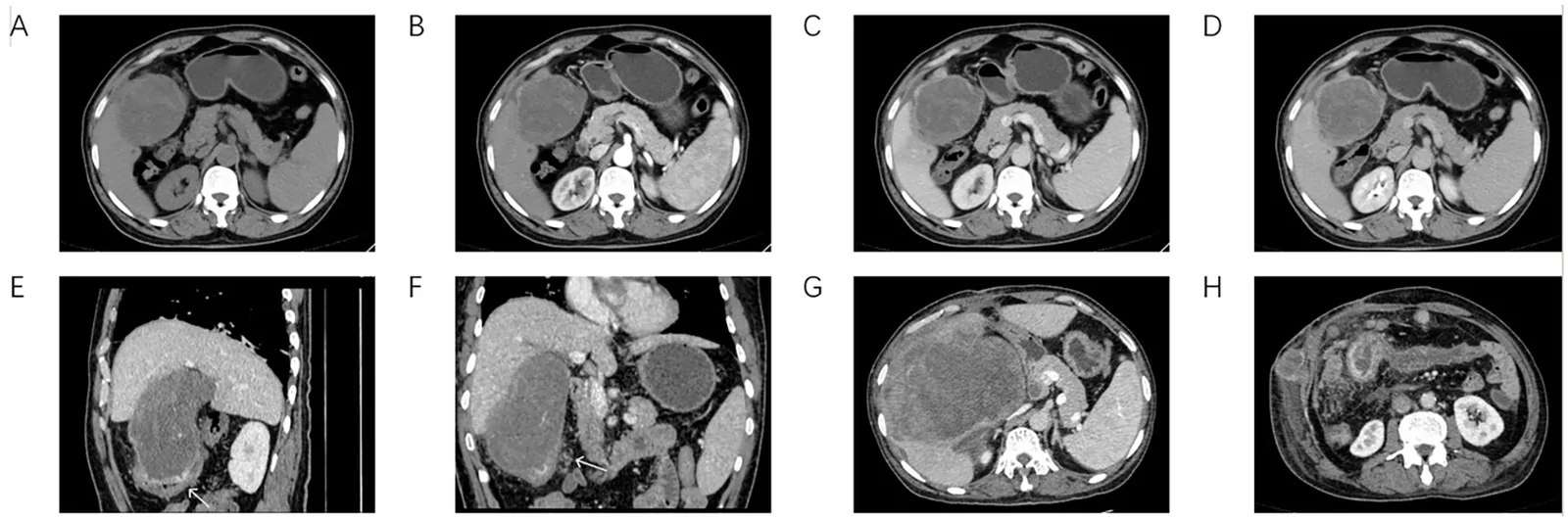
**(A)** Non-contrast CT scan showing an enlarged gallbladder with patchy soft tissue density shadows within; **(B)** Contrast-enhanced arterial phase; **(C)** Contrast-enhanced venous phase; **(D)** Contrast-enhanced delayed phase; **(B–D)** demonstrate patchy, progressive enhancement of the intra-gallbladder lesion; **(E)**. Contrast-enhanced scan, venous phase, sagittal reconstruction: blurred fat planes at the arrow indicate tumour invasion into the hepatic flexure of the colon; **(F)**. Contrast-enhanced scan, venous phase, coronal reconstruction: multiple small vessels clustered at the arrow; **(G)**. Follow-up examination 2 months post-operatively: massive mass shadow visible in the gallbladder fossa; **(H)**. Multiple soft tissue nodules noted in the right subcutaneous space of the lower abdomen and the right omentum.

Contrast-enhanced CT of the upper abdomen revealed a markedly enlarged gallbladder with irregular wall thickening. Multiple linear and patchy isodense lesions were observed within the gallbladder lumen, most prominently at the gallbladder base, with attenuation values ranging from 20.5 to 42.3 HU. Scattered punctate hypodense foci were also noted intraluminally. On enhanced imaging, the lesions within the gallbladder lumen exhibited progressive, non-uniform enhancement. Furthermore, the interface between the gallbladder wall and the adjacent liver parenchyma, as well as the hepatic flexure of the colon, appeared indistinct. These findings suggested a space-occupying lesion within the gallbladder lumen accompanied by multiple gallstones (**Figures 1A–F**).

**Figure 2:**
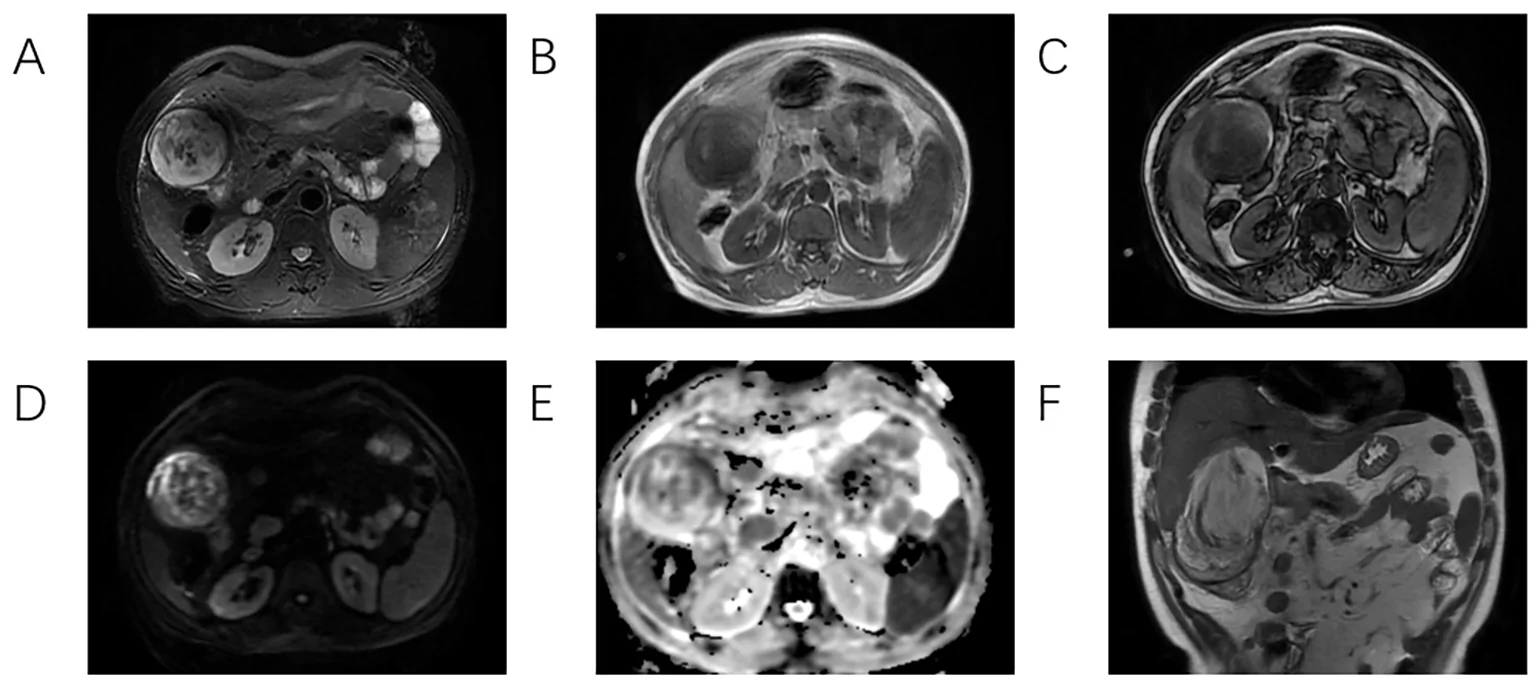
**(A)** T2WI: The gallbladder cavity exhibits mixed signal intensity with patchy isointense to hypointense areas of irregular morphology. **(B)** T1WI: The lesion demonstrates isointense to hypointense signal. **(C)** T1WI (reverse phase): No signal reduction observed in the lesion. **(D)** DWI: Patchy and punctate hyperintense signals are visible within the gallbladder lumen. **(E)** ADC: Signal reduction in the lesion. **(F)** T1W1 coronal view: Indicates tumour invasion into the hepatic flexure of the colon.

MRI demonstrated an enlarged gallbladder with irregular wall thickening and heterogeneous intraluminal signal intensity. Multiple cord-like areas of low signal intensity were observed on T2-weighted images. Additionally, the gallbladder lumen exhibited streaky and punctate hyperintense areas on diffusion-weighted imaging (DWI) with corresponding low signal intensity on apparent diffusion coefficient (ADC) maps. The interface between the tumor and the hepatic flexure of the colon remained indistinct (**Figure 2**).

Based on the preoperative diagnosis of a gallbladder mass accompanied by gallstones, the patient underwent a radical cholecystectomy under general anesthesia. Intraoperative findings revealed a markedly enlarged gallbladder with congestion and edema.The pericholecystic fluid exhibited purulent inflammatory changes, along with dense adhesions to the greater omentum, colon, and duodenum. Following meticulous adhesiolysis, the gallbladder wall appeared thickened and fragile with a rupture identified through which dark brown, “fish-flesh–like” tumor tissue and multiple stones were discharged. The tumor demonstrated direct invasion into the hepatic flexure of the colon. Consequently, the lesion was resected en bloc with the adjacent omentum and a portion of the colonic wall, followed by primary repair of the colon. The patient developed postoperative intestinal obstruction and subsequently required a secondary ileostomy.( The patient developed postoperative intestinal obstruction and subsequently underwent reoperation with creation of an end ileostomy).

**Figure 3:**
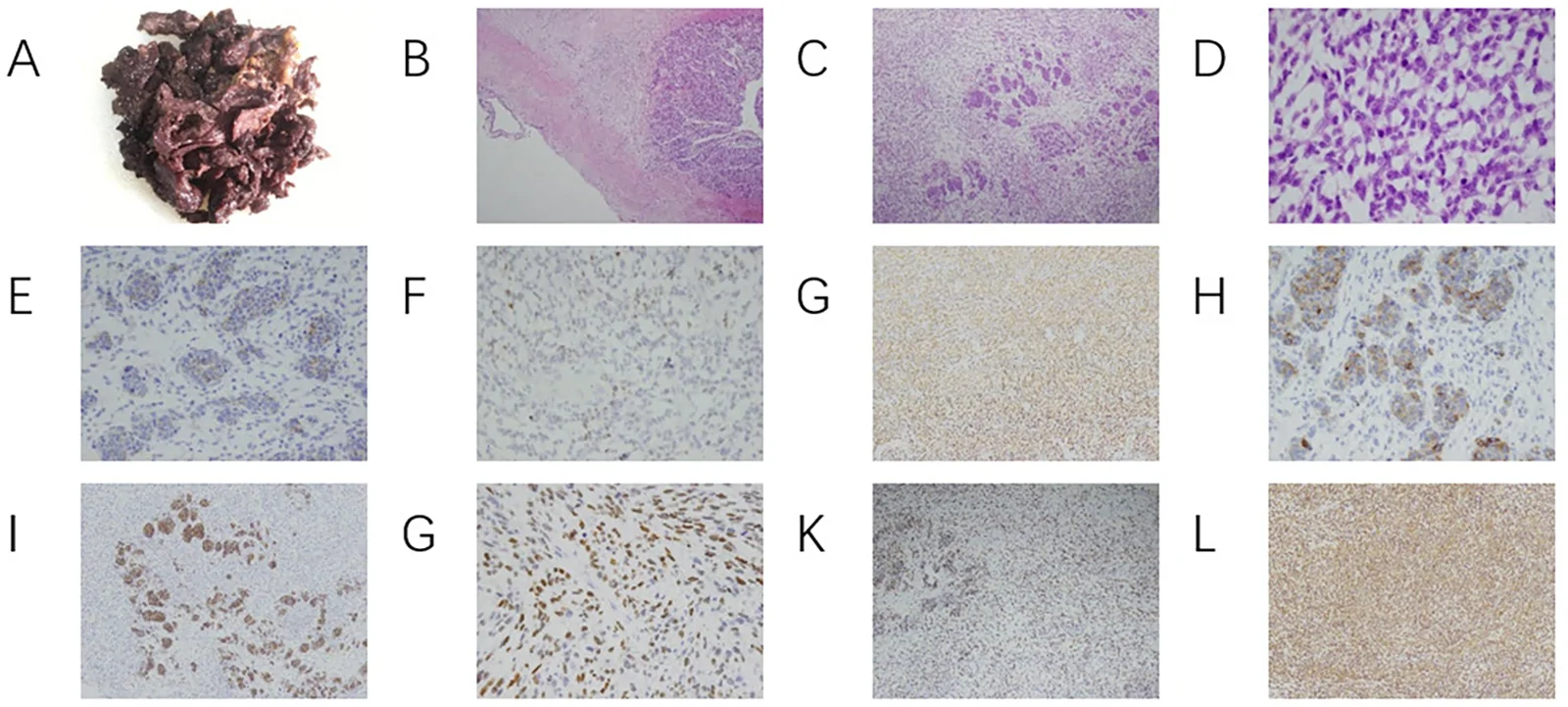
**(A)** Remaining tissue following the collection of pathological examination specimens; **(B)** Tumour invading nearly the entire gallbladder wall, with only partial mucosa remaining (HE, x40); **(C)** Localised squamous metaplasia in tumour cells (HE, x100); **(D)** Moderate to severe atypia in tumour cells with visible pathological mitotic figures (HE, x600); **(E)** Focal CK-pan positivity in tumour cells (HE, x400); **(F)** Focal S-100 positivity in tumour cells (HE, x400); **(G)** Focal SMA positivity in tumour cells (HE, x100); **(H)** tumour cells EMA focal positive (HE, x400); **(I)** tumour cells CD117 focal positive (HE, x100); **(G)** tumour cells p63 focal positive (HE, x400); **(K)**: Ki-67 proliferation index approximately 60% (HE, x100); **(L)**: Tumour cells diffusely positive for vimentin (HE, x100).

Postoperative histopathological examination revealed destruction of the gallbladder architecture, with mucosal ulceration and a large amount of dark brown fragmented tissue measuring 13 × 13 × 3 cm. The tumor infiltrated almost the full thickness of the gallbladder wall, with extensive hemorrhage and necrosis. The tumor cells were spindle-shaped or epithelioid, with focal squamous metaplasia, and were arranged in cord-like patterns. The stroma showed myxoid changes, with moderate to severe cellular atypia and numerous mitotic figures, including atypical mitoses (**Figures 3A–D**).

Immunohistochemical analysis demonstrated focal positivity for CK-pan, S-100, SMA, EMA, CD117, and p63 (**Figures 3E–G**), while CD34, desmin, calponin, brachyury, GFAP, and TLE1 were negative. The Ki-67 proliferation index was approximately 60%, and the tumor cells exhibited diffuse positivity for vimentin (**Figures 3K, L**). The final pathological diagnosis was primary MC of the gallbladder.

Two months postoperatively, follow-up CT revealed tumor recurrence, characterized by a large mass in the gallbladder fossa with heterogeneous density and indistinct margins. Contrast-enhanced imaging demonstrated heterogeneous enhancement with invasion into the adjacent liver parenchyma. Furthermore, multiple soft tissue nodules were identified within the subcutaneous fat of the right lower abdomen and the omentum, exhibiting annular enhancement. Blurring of the peritoneal fat planes was also observed (**Figures 1G–H**), suggesting multiple metastases to the peritoneal cavity and subcutaneous tissue, alongside involvement of the hepatic flexure of the colon. The patient succumbed to the disease three months postoperatively.

## Discussion

MC of soft tissue is an exceptionally rare and highly aggressive malignancy. A systematic search of the PubMed and CNKI databases over the past two decades identified only five documented cases of primary MC arising from visceral soft tissues, involving the heart (n=3), stomach (n=1), and kidney (n=1). To the best of our knowledge, the current case represents the sixth reported instance of visceral involvement, and the first to originate in the gallbladder. These cases are summarized in **Table 1**.

**Table 1:** MC arising from visceral soft tissues.

Project	Case 1 Gleason et al. (2007) (4)	Case 2 Tseng et al. (2015) (5)	Case 3 Patel et al. (2019) (6)	Case 4 Zhao Bin et al. (2021) (7)	Case 5 Mwizerwa et al. (2025) (8)	Case 6 This case
Gender/Age	Female / 15 years old	Female / 61 years old	Male / Four months	Male / 63 years old	Male/12 years old	Male / 57 years old
Site of onset	Heart	Stomach	Heart	Right kidney	Heart (left ventricle)	Gallbladder
Tumour size	6.5 cm	1.3 cm	4.3cm	6.0 cm	7.3cm	2.5 cm
Imaging Findings	Not provided	Localized thickening of the gastric wall was observed, with no evidence of lymph node enlargement or distant metastasis.	The margins were indistinct. On T1- and T2-weighted images, the lesion showed signal intensity similar to that of the myocardium. On contrast-enhanced imaging, it demonstrated lower enhancement than the myocardium.	A nodular lesion was observed in the right renal pelvis, with thickening of the upper segment of the right ureter and mild hydronephrosis of the right kidney.	The lesion demonstrated high signal intensity on both T1- and T2-weighted images, with delayed enhancement.	CT demonstrated irregular thickening of the gallbladder wall and multiple linear or band-like isodense areas within the lumen, showing progressive, heterogeneous enhancement after contrast administration. MRI demonstrated multiple linear or band-like areas within the gallbladder lumen that were iso- to hypointense on T2-weighted images and hyperintense on diffusion-weighted imaging (DWI).
Molecular testing	EWSR1 rearrangement negative (in some cases)	EWSR1 rearrangement negative	EWSR1 rearrangement positive	EWSR1 rearrangement positive	EWSR1 rearrangement positive	Not detected
Immunohistochemistry positive	CK+, EMA+, S-100+, GFAP+	Vim+, S-100+, CK+, EMA+, Syn+ DOG1+, CD10+ (focal)	S-100+, AE1/AE3+, EMA+, GFAP+, INI-1 retention	Vim+, SMA+, CK+, EMA+, p63+ INI-1 negative, Ki-67 40%	S-100+, AE1/AE3+, EMA+, SOX10, HHF35,	Vim+, CK-pan+, S-100+, SMA+, EMA+, CD117+, p63+ INI-1 preserved, Ki-67 60%
Surgical procedure	Biopsy and chemotherapy (unresectable)	Local excision	Subtotal resection of the right ventricular outflow tract tumor with right ventricular outflow tract reconstruction	Right nephrectomy	Tumour resection with mitral valve repair	Radical cholecystectomy with partial colectomy and ileostomy
Pronostic	Death (metastasis to the lungs)	No recurrence/ metastasis (10-month follow-up)	Brain metastases developed 16 months post-surgery; death occurred 22 months post-surgery.	No recurrence/ metastasis (under follow-up)	No recurrence/ metastasis (under follow-up)	Death within three months of surgery
Pathological diagnosis	Cardiac myoepithelial carcinoma	Low-grade gastric myoepithelioma	Cardiac myoepithelial carcinoma	Renal myoepithelial carcinoma	Cardiac myoepithelial carcinoma	Gallbladder myoepithelial carcinoma

Clinically, soft tissue MC lacks specific clinical manifestations. While tumors in the head and neck region may manifest as rapidly enlarging over a short period, fixed masses associated with tenderness or facial nerve palsy (9), visceral tumors typically present with non-specific symptoms related to mass effect and local compression. In the present case, the patient’s primary complaint was abdominal pain. The absence of specific clinical indicators frequently hinders early diagnosis, which may potentially contribute to the unfavorable prognosis of this disease.

Histopathological examination remains the definitive diagnosis modality for MC of gallbladder. Histologically, soft tissue MC is characterized by tumor cells consisting predominantly of myoepithelial cells, which exhibit a spectrum of clear-cell, epithelioid, or spindle-shaped morphologies. These cells typically feature abundant cytoplasm, significant nuclear atypia, and frequent mitotic figures. The tumor typically exhibits an infiltrative growth pattern with ill-defined margins and the absence of a well-defined capsule. Immunohistochemically, MC characteristically expresses both epithelial and myogenic markers, reflecting biphasic differentiation. Tumor cells typically show positivity for epithelial markers such as cytokeratin (CK) and epithelial membrane antigen (EMA), supporting epithelial differentiation (2). Furthermore, the vast majority of cases express at least one myoepithelial marker, such as S-100, SMA, or p63, supporting myoepithelial differentiation (2). In the present case, the tumor diffusely infiltrated the gallbladder wall, leaving only minimal residual mucosa. The tumor cells exhibited diverse morphologies, predominantly spindle-shaped and epithelioid, with marked nuclear atypia and numerous atypical mitotic figures. The cells were mainly arranged in cords, with focal squamous metaplasia, and the stroma showed myxoid changes. Immunohistochemistry demonstrated co-expression of epithelial markers, including vimentin, CK-pan, and EMA, as well as multiple myoepithelial-associated markers (eg. S-100, SMA, CD117, and p63). Collectively, these morphological and immunophenotypic hallmarks established the definitive diagnosis of primary MC of the gallbladder.

According to the previous literature, myoepithelial tumors of soft tissues-particularly those in the head and neck region-typically manifest as isointense or hypointense on T1-weighted images and isointense to hyperintense on T2-weighted images (10). Most tumors are invasive and may metastasize to distant sites (11), with MC demonstrating a relatively higher metastatic potential (3).On non-contrast CT, MC of soft tissue usually present as heterogeneous masses with ill-defined margins and a mean attenuation value of approximately 40 Hounsfield Units (HU) (12, 13). Upon contrast-enhanced CT, these tumors typically demonstrate heterogeneous enhancement, with mild-to-moderate enhancement in the arterial phase and persistent enhancement in the venous phase (14).In the present case, CT findings of irregular gallbladder wall thickening and multiple linear or band-like isodense intraluminal lesions were broadly consistent with these documented imaging hallmarks. MRI revealed linear or band-like areas of low signal intensity on T1-weighted images and iso- to hypointense signal on T2-weighted images, corresponding to the postoperative pathological finding of multiple tissue fragments within the gallbladder.

Furthermore, a broad spectrum of conditions can lead to gallbladder wall thickening, encompassing inflammatory disorders such as xanthogranulomatous cholecystitis (XGC), neoplastic malignancies like gallbladder carcinoma (GBC), and proliferative lesions such as gallbladder adenomyomatosis. XGC and gallbladder adenomyomatosis typically exhibit characteristic imaging features, such as the “sandwich sign” and Rokitansky–Aschoff sinuses (15–17), consequently, they are relatively distinguishable from MC of the gallbladder. However, as both GBC and MC are aggressive malignancies that exhibit significant radiological overlap, their preoperative differentiation on imaging remains highly challenging.

GBC is the most prevalent primary malignanancy of the biliary tract. Conventional gallbladder adenocarcinomas typically express epithelial markers, such as CK7, CK19, and CEA (18), while lacking expression of myogenic markers. Therefore, a dual-positive immunophenotype has significant specificity and diagnostic value for the diagnosis of primary MC of the gallbladder.

On non-contrast CT, GBC typically appears as a mass of iso- to slightly low attenuation within the gallbladder, frequently accompanied by irregular thickening of the gallbladder wall. Contrast-enhanced imaging demonstrates a “fast-in, slow-out” pattern, characterized by irregular enhancement in the arterial phase and persistent enhancement in the portal venous phase (19), with marked enhancement of both the tumor and adjacent gallbladder wall. This enhancement pattern differs from that observed in MC and may aid in differentiating between the two. On MRI, GBC typically appears hypointense on T1-weighted images and slightly hyperintense on T2-weighted images, with restricted diffusion on diffusion-weighted imaging (DWI) (20). These MRI characteristics diverge significantly from the features identified in the present case. Furthermore, GBC usually presents as a solid mass (21), whereas the present case demonstrated distinctive cord-like structures resembling “fish flesh” on both imaging and gross pathological examination. This unique morphological hallmark may represent a pivotal diagnostic criterion for primary MC of the gallbladder.

In conclusion, we report the first documented case of primary myoepithelial carcinoma of the gallbladder, a rare malignancy characterized by non-specific clinical manifestations but distinctive imaging features. On CT, the lesion appears as an irregular, heterogeneously attenuating, band-like intraluminal mass exhibiting progressive, non-uniform enhancement. MRI further demonstrates a band-like intraluminal lesion with predominantly low signal intensity on both T1- and T2-weighted images, accompanied by heterogeneous signal characteristics, and appears hyperintense on diffusion-weighted imaging (DWI).In clinical practice, radiologists should be familiar with the imaging characteristics of MC arising from visceral soft tissues. When encountering a mass with heterogeneous attenuation, ill-defined margins, progressive enhancement, and predominantly low signal intensity on both T1- and T2-weighted images, along with cord-like low-signal areas, primary MC of visceral origin should be considered in the differential diagnosis to ensure timely and appropriate clinical management.

## Data Availability

The raw data supporting the conclusions of this article will be made available by the authors, without undue reservation.
